# Ultrafast terahertz snapshots of excitonic Rydberg states and electronic coherence in an organometal halide perovskite

**DOI:** 10.1038/ncomms15565

**Published:** 2017-06-01

**Authors:** Liang Luo, Long Men, Zhaoyu Liu, Yaroslav Mudryk, Xin Zhao, Yongxin Yao, Joong M. Park, Ruth Shinar, Joseph Shinar, Kai-Ming Ho, Ilias E. Perakis, Javier Vela, Jigang Wang

**Affiliations:** 1 Division of Materials Science and Engineering, Ames Laboratory, Iowa State University, Ames, Iowa 50011, USA; 2 Department of Physics and Astronomy, Iowa State University, Ames, Iowa 50011, USA; 3 Department of Chemistry, Iowa State University, Ames, Iowa 50011, USA; 4 Department of Physics, University of Alabama at Birmingham, Birmingham, Alabama 35294-1170, USA; 5 Division of Chemical and Biological Sciences, Ames Laboratory, Iowa State University, Ames, Iowa 50011, USA

## Abstract

How photoexcitations evolve into Coulomb-bound electron and hole pairs, called excitons, and unbound charge carriers is a key cross-cutting issue in photovoltaics and optoelectronics. Until now, the initial quantum dynamics following photoexcitation remains elusive in the hybrid perovskite system. Here we reveal excitonic Rydberg states with distinct formation pathways by observing the multiple resonant, internal quantum transitions using ultrafast terahertz quasi-particle transport. Nonequilibrium emergent states evolve with a complex co-existence of excitons, carriers and phonons, where a delayed buildup of excitons under on- and off-resonant pumping conditions allows us to distinguish between the loss of electronic coherence and hot state cooling processes. The nearly ∼1 ps dephasing time, efficient electron scattering with discrete terahertz phonons and intermediate binding energy of ∼13.5 meV in perovskites are distinct from conventional photovoltaic semiconductors. In addition to providing implications for coherent energy conversion, these are potentially relevant to the development of light-harvesting and electron-transport devices.

## Abstract (web summary)

The generation of bound electron and hole pairs—excitons—is a key process in photovoltaic technologies, yet it is challenging to follow their initial dynamics. Here, Luo *et al*. probe the Rydberg eigenstates that characterize the excitonic transport and coherent conversion in a perovskite material.

The initial pathways for the photoconversion into exciton populations rely on two dynamic processes mediated by carrier–carrier and –phonon interactions^1^,^2^: quantum dynamics leading to conversion of electronic coherence into incoherent populations following polarization dephasing, and cooling of hot states via phonon-assisted energy loss and momentum transfer. In organic photovoltaic systems, the prevailing picture is that photo-generated excitons quasi-instantaneously form during the extremely fast ∼10 s of fs dephasing times and, then, on a similar time scale, substantially populate interfacial, charge transfer states as *e*-*h* polaron pairs. Because of the limiting long-range charge separation and transfer inherent in such polaron formation, reexamination of the conventional wisdom is being actively pursued in efficient photoconversion systems. Of particular interest are the emerging concepts of quantum coherent transport^3^,^4^ and hot-state delocalization^5^ on ultrafast time scales. In the hybrid perovskite materials, such fundamental ultrafast photoconversion pathways and electronic coherence are expected to be even more important but have not been studied. This limits a thorough understanding of the fundamental photoconversion mechanism relevant for device applications^6^,^7^,^8^,^9^,^10^,^11^.

The energy spectrum of Coulomb-bound excitons, within a 1D hydrogen atom-like description, is characterized by a series of discrete bright (1*s*, 2*s ...*) and optically-forbidden, dark Rydberg states (2*p*, 3*p...*), as illustrated in Fig. 1a near centre-of-mass momentum *K*≈0. It has been well established that exciton binding energies are in the range of few meV for inorganic bulk semiconductors due to the small effective mass and large dielectric screening, while substantially larger binding energies of 100 s of meV arise in organic semiconductors^3^,^4^,^5^. In the hybrid organic–inorganic perovskites, however, the determination of exciton binding energy remains challenging. Thus far, the scattered experimental results are intensely debated and show a large inconsistency in the reported values of 2–50 meV (5–62 meV) at low (room) temperatures^12^,^13^,^14^,^15^,^16^,^17^,^18^,^19^,^20^. While the previous magneto-optical and light absorption measurements have established the existence of spectral features of bright excitons^12^,^13^,^14^,^15^,^16^,^17^,^18^,^19^,^20^, the higher-lying, dark Rydberg states, another equally important hallmark, have not been established. In addition, the previous approaches provide no insight into the exciton populations and dynamics, since they measure linear responses determined by the optical polarization and not the populations^1^. (See also Supplementary Note 1).

Ultrafast terahertz (THz) spectroscopy provides an unambiguous approach to characterize full response functions of genuine excitons and carriers, as well as their dynamics. Broadband THz pulses probe discrete, internal quantum transitions between bright and dark excitonic Rydberg eigenstates such as 1*s*→2*p* and 1*s*→3*p*, as illustrated in Fig. 1a, which provide a direct measure of excitonic correlations and binding energies^21^,^22^,^23^. Pump-induced THz conductivity is sensitive to excitons across the entire centre-of-mass momentum *K*-space. In contrast, the previously-used interband optics only measures a subset of excitons near *K*=0. In addition, controllable initial conditions, determined by electronic coherence, hot excitons and the carrier plasma, can be selectively generated and studied by on- or off-resonant laser pumping. Before THz experiments on perovskite materials have focused on effects such as Auger recombination and phonons in the range of 2–10 meV (refs 13, 24, 25, 26, 27). A recent broadband measurement have been performed only at room temperature, tetragonal phase and under very high pumping with transient carrier density above the Mott transition^14^. Large thermal broadening and dielectric screening^27^,^28^ are likely to prevent it from providing information on the exciton formation pathways and initial quantum dynamics.

Here we report the direct observation of resonant, internal quantum transitions between excitonic Rydberg states and their distinct formation pathways in a model perovskite CH_3_NH_3_PbI_3_ (or MAPbI_3_) by measuring the sub-ps resolved, full THz response functions. This precisely yields a binding energy 13.5 meV that appears exclusively below the tetragonal-to-orthorhombic structural phase transition at *T*_*S*_=160 K. Nonequilibrium emergent states evolve with a complex co-existence of excitons, carriers and phonons, where a delayed buildup of excitons under various pumping conditions allows us to distinguish between the loss of electronic coherence and hot state cooling processes. Such initial coherent and cooling dynamics of excitons further elucidate the nearly ∼1 ps dephasing time and strong interaction between electron and low-frequency phonons in perovskites that are distinct from conventional semiconductors. In addition to providing implications for coherent energy conversion, these fundamental insights are potentially relevant to the development of light-harvesting and electron-transport devices.

## Results

### Experimental scheme and sample characterization

**Figure 1:**
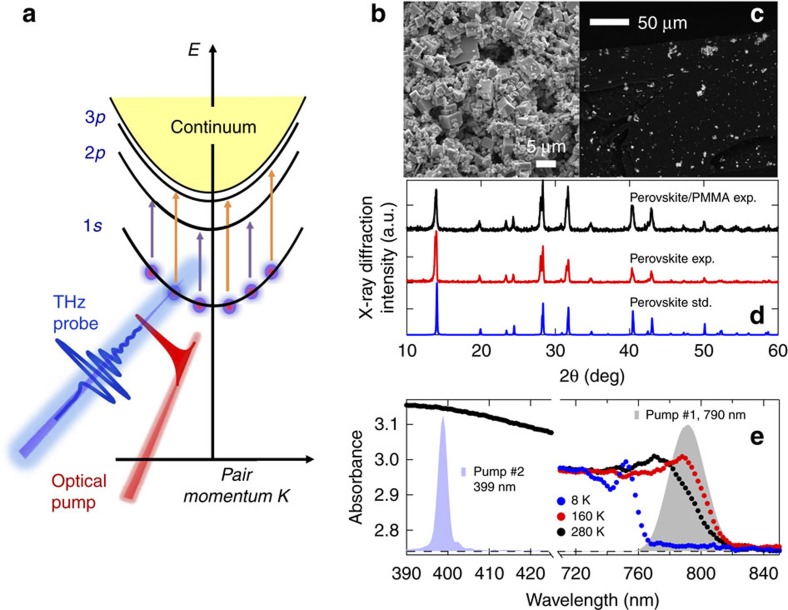
THz conductivity measurement scheme and sample characterizations. (**a**) Schematics of exciton dispersion near *K*≈0, with internal quantum transitions of 1*s*→2*p* and 1*s*→3*p* marked. Photoexcitation creates pair transitions in the perovskites that are resonantly probed by THz pulses. (**b**,**c**) SEM images of pure CH_3_NH_3_PbI_3_ powder and CH_3_NH_3_PbI_3_/PMMA film, respectively. (**d**) X-ray diffraction measurement of perovskite film and powder. (**e**) Absorption spectra of the perovskite/PMMA film at various temperatures. Shown together are the two pump spectra (#1 and #2, shade).

A 550 μm thick free standing perovskite-poly(methylmethacrylate) (PMMA) thin film was made by embedding μm size CH_3_NH_3_PbI_3_ crystals (Fig. 1b) in the polymer matrix, as shown in SEM image of the CH_3_NH_3_PbI_3_/PMMA film (Fig. 1c). Room temperature X-ray diffraction measurements of the film and pure CH_3_NH_3_PbI_3_ powder corroborate the inclusion of the perovskites into the PMMA matrix (Fig. 1d). We also performed comprehensive temperature-dependent absorption (Fig. 1e), photoluminescence and X-ray diffraction measurements of our samples down to *T*=6 K (Supplementary Figs 1–3), which are consistent with prior studies of high-quality perovskite materials^12^,^13^,^18^. In addition, there is no evidence for the presence of any tetragonal impurities at low temperature, for example, 8 K at which most of our experiments are performed. Note the absorption curves in Fig. 1e display very big light scattering as manifested by the large background above 820 nm, which is below the perovskite band gap and little absorption is expected. We attribute this to the high inhomogeneity of perovskite crystals dispersed in PMMA matrix, as confirmed in ref. 29 (See Supplementary Note 4 for details).

**Figure 2:**
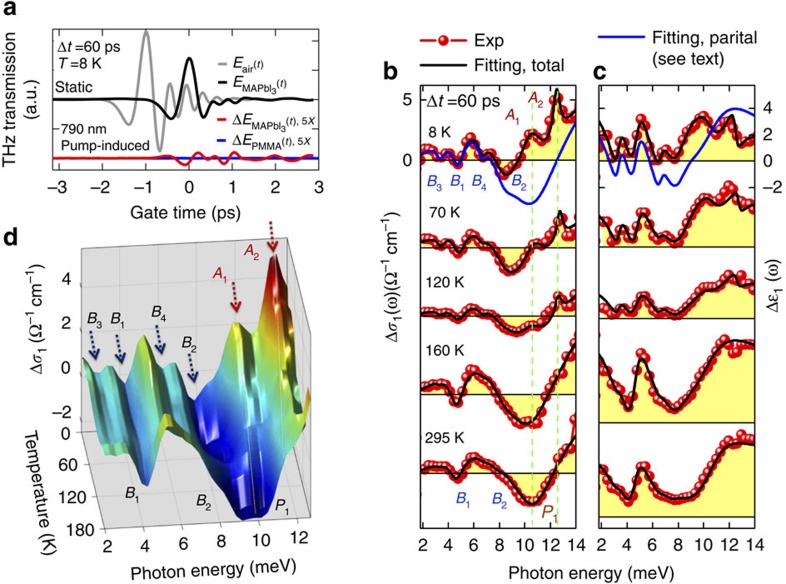
The direct observation of excitonic Rydberg states in CH_3_NH_3_PbI_3_. (**a**) THz fields transmitted (raw data) through a clear aperture (grey), the sample without pump (black) and its pump-induced change (red) under 790 nm wavelength and 550 μJ cm^−2^ fluence, at *T*=8 K and pump probe delay Δ*t*=60 ps. There is negligible pump-induced change for pure PMMA (blue). (**b**,**c**) Ultrafast THz spectra Δ*σ*_1_(*ω*) and Δ*ɛ*_1_(*ω*) under the same pumping conditions as (**a**) for various temperatures. This demonstrates the distinct resonant, internal quantum transitions, the 1*s*→2*p* and 1*s*→3*p* marked as *A*_1_ and *A*_2_, which are clearly different from the phonon bleachings in the CH_3_NH_3_PbI_3_ (*B*_*j*=1,..,4_) and PMMA (*P*_*k*=1,2_) (Supplementary Fig. 4 and Supplementary Note 4). Shown together are the complete (black lines) and partial (blue lines, without excitonic contribution) model calculations by equation (1). (**d**) A 3D view of the temperature-dependent Δ*σ*_1_(*ω*) spectra further confirms the fine details of the phonon bleaching modes.

We perform optical-pump and THz-probe spectroscopy, which is driven by a 1 kHz Ti:Sapphire regenerative amplifier with 790 nm central wavelength and 40 fs pulse duration^22^,^23^,^30^,^31^. One part of the output is used to pump the sample either directly at the fundamental wavelength of 790 nm or at 399 nm after going through a *β*-barium-borate (BBO) crystal. The pump spectra are shown in Fig. 1e together with the linear absorption spectra of the CH_3_NH_3_PbI_3_/PMMA film. The other part of the output is used to generate and detect phase-locked THz fields in time-domain via optical rectification and electro-optic sampling of two 0.2 mm thick ZnTe crystals, respectively. This increases by an order of magnitude of the signal-to-noise ratio at the important, high frequency regions in the range of 10–15 meV comparing to 1 mm thick ZnTe emitter/detector. The THz electrical fields exhibit broadband spectral width from 2 to 15 meV used as a probe. To obtain time-resolved complex THz conductivity of the sample, we record the THz electrical fields in time-domain transmitted through a clear aperture *E*_air_(*t*), static sample 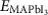
(*t*) and its pump-induced change Δ
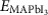
(*t*) at a fixed pump-probe delay Δ*t* (Fig. 2a). Note there is negligible pump-induced change for a pure PMMA control sample Δ*E*_PMMA_(*t*).

### Correlated THz resonances from excitonic Rydberg states

Figure 2a allows us to simultaneously obtain the sub-ps, frequency-dependent complex conductivity Δ*σ*_1_(*ω*) (Fig. 2b) and dielectric function Δ*ɛ*_1_(*ω*) (Fig. 2c), associated with dissipative and inductive responses of the sample^31^ (also see Methods and Supplementary Note 3). At high temperature above the structural phase transition at *T*_*S*_=160 K, the 295 K trace exhibits two strong photoinduced bleaching features, that is, negative conductivity (Δ*σ*_1_<0), at 4.2 and 10 meV. These arise from pump-induced softening of phonon modes in CH_3_NH_3_PbI_3_ at *B*_1_∼4.2 meV, *B*_2_∼8 meV, and in PMMA at *P*_1_∼10 meV (see Supplementary Fig. 4 and Supplementary Note 4). The last two merge into a broad bleaching mode. Below *T*_*S*_ two new bleaching modes become progressively pronounced, as marked in the 8 K trace at *B*_3_∼3 meV and *B*_4_∼6.2 meV, fully consistent with prior observations^13^,^24^,^25^. However, remarkable photoinduced absorptive resonances (Δ*σ*_1_>0), previously unobserved, appear at frequencies above 10 meV, but only below *T*_*S*_. Most intriguingly, the pronounced absorptive resonances, centred at *A*_1_∼10.1 meV and *A*_2_∼12.1 meV, completely prevail over the bleaching behaviours, as seen in the 8 K trace (Fig. 2b). Figure 2d further shows the resonant absorption significantly increases with decreasing temperature, which cannot be attributed to the phonon bleaching. Moreover, the high energy spectral shapes in Δ*σ*_1_ and Δ*ɛ*_1_ are characterized by the correlated, dissipative and inductive features of two well-defined *A*_1_ and *A*_2_ resonances. These point to the internal transitions between Rydberg states, that is, 1*s*→2*p* and 1*s*→3*p*. We emphasize that the experimental ratio of the oscillator frequencies *A*_1_/*A*_2_=0.835 is in perfect agreement with the theoretical value *ω*_1*s*→2*p*_/*ω*_1*s*→3*p*_=(3/4)/(8/9)=0.844 derived from the quantized bound states *E*_n_=−*E*_1*s*_/*n*^2^, where 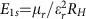. *μ*_*r*_, *ɛ*_*r*_ and *R*_*H*_ are the relative effective mass, relative permittivity and Rydberg constant, respectively. This yields exciton binding energy *E*_1*s*_=13.5 meV. In addition, the disappearance of the resonances across *T*_*S*_ indicates a sudden drop in *E*_1*s*_ below ∼2 meV, our low-frequency resolution, consistent with the tetragonal-to-orthorhombic transition^27^,^28^.

To put our observation of resonant quantum transitions of excitonic Rydberg states on a solid footing, we fit the experimentally-determined, sub-ps THz response function by a model that consists of the 1*s*→*np* (*n*=2,3) excitonic resonances, the ‘local' resonant phonon bleaching and Drude–Smith responses from disorder-/backscattering-induced transport of unbound carriers (see Methods). The experimentally-determined, sub-ps THz response functions can be fitted by a model that assumes co-existence of excitons, phonons and *e*-*h* plasma (see Supplementary Note 4):


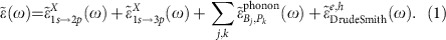


The first two terms describe the 1*s*→*np* (*n*=2,3) excitonic resonances which are proportional to the effective transition strength 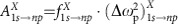, where *f* is the intra-excitonic oscillator strength, and the plasma frequency 
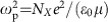
. *N*_*X*_ is the exciton density. This way, 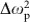 is proportional to the population difference between the two Rydberg states involved in the transitions (Fig. 1a), that is, Δ*N*_1*s*,*np*_=*N*_1*s*_−*N*_*np*_ (ref. 22). Next, the ‘local' resonant bleaching features are well represented by the third term in equation (1), the sum of the phonon contributions in the CH_3_NH_3_PbI_3_ (*B*_*j*=1,..,4_) and PMMA (*P*_*k*=1,2_) (see Supplementary Fig. 4 and Supplementary Note 4). Lastly, the non-resonant component is described by the fourth Drude–Smith term in equation (1), which describes disorder-/backscattering-induced transport of unbound carriers of density *N*_*eh*_^32^,^33^. The last term is justified from the suppressed Δ*σ*_1_(*ω*) (Fig. 2b) and rapidly increased Δ*ɛ*_1_(*ω*) (Fig. 2c) as *ω*→0, in contrast to the conventional Drude transport of unbound *e*-*h* carriers. The Drude-Smith term is proportional to 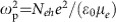.

Both Δ*σ*_1_(*ω*) and Δ*ɛ*_1_(*ω*) can be consistently fitted very well over the entire spectral, up to 14 meV, and temperature ranges (black solid lines in Fig. 2b,c). This is remarkable considering that the fit are strongly restrained by the requirement of simultaneously describing both responses over a broad spectral range, and by the distinctly different spectral shapes of the excitons and charge carriers. Therefore, by fitting the experimentally obtained THz response functions with the theoretical model, we are able to calculate the densities of excitons Δ*N*_1*s*,*np*_ and unbound charge carriers *N*_*eh*_, as shown in the discussion below.

### Excitonic formation pathways characterized by THz responses

**Figure 3:**
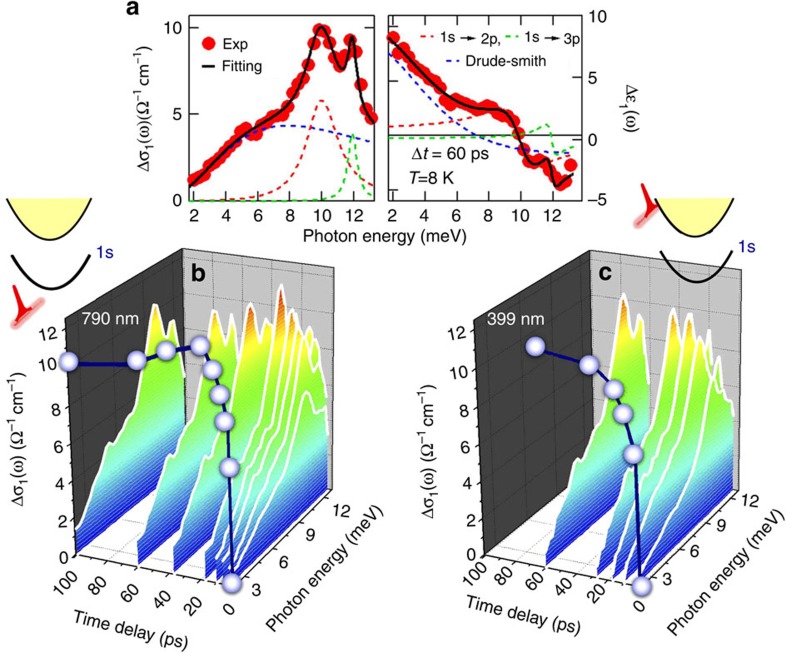
Ultrafast THz Snapshots of formation pathways of excitonic Rydberg states. (**a**) The THz response functions (red dots) measured under 790 nm wavelength and 550 μJ cm^−2^ fluence, at *T*=8 K and pump probe delay Δ*t*=60 ps. The fit (black lines), based on the analytical model of equation (1), is the sum of 1*s*→2*p* (dashed red lines), 1*s*→3*p* (dashed green lines) and unbound *e-h* carriers (dashed blue lines). (**b**,**c**) Photoinduced conductivity changes Δ*σ*_1_(*ω*) at several pump-probe delays and *T*=8 K after excitation at 790 nm (550 μJ cm^−2^ fluence) and 399 nm (120 μJ cm^−2^ fluence), respectively. The shaded circles are the effective transition strength 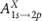, extracted from photoinduced internal quantum transitions of excitons. The pump fluences are chosen to induce approximately equivalent 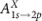 for both excitations which allows to underpin their distinctly different rise times.

To further investigate the buildup of the internal quantum transitions, Fig. 3a highlights the complete characterization of full response functions from three electronic contributions at 8 K after removing the phonon contributions, that is, photo-generated excitons 1*s*→2*p* (dashed red lines), 1*s*→3*p* (dashed green lines) and from unbound *e-h* carriers (dashed blue lines). This has not been possible in prior measurements in the perovskites. Such spectra are plotted in Fig. 3b for various time delays Δ*t* under 790 nm excitation at 550 μJ cm^−2^, *T*=8 K, that is, for pump tuned slightly below the lowest bound, 1*s* exciton (pump #1, Fig. 1e). For such below-resonance pumping we expect to mainly generate electronic coherence at early times with minimum heating since only absorption appears at the high energy tail of the pump spectrum. Remarkably, the 1*s*→2*p* and 1*s*→3*p* quantum transitions in Δ*σ*_1_(*ω*) exhibit a delayed rise persisting up to ∼17 ps. In strong contrast, excitation of the higher energy, *e*-*h* continuum under 399 nm at 120 μJ cm^−2^, *T*=8 K (pump #2, Fig. 1e) gives rise to much longer buildup times of 10s of ps (Fig. 3c). Such continuum, *e*-*h* excitations are unbound and thus expected to lose electronic coherence quasi-instantaneously following the fs pulse, which leads to a hot electron distribution. An excitonic 1*s* population can then be formed only when the continuum of phonons are scattered to cool the hot state. Such a process is slowed down by the requirement of many scattering events and hot-phonon effects^34^.

### Time-dependent densities of excitons and unbound carriers

**Figure 4:**
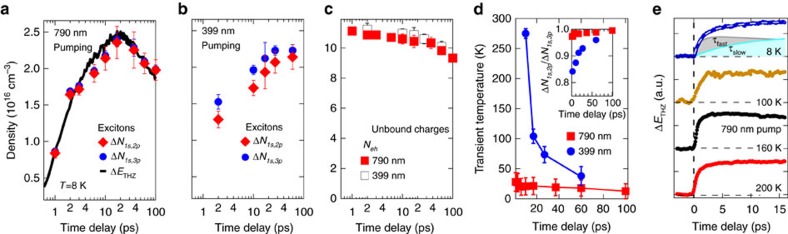
The time-evolution of the exciton distribution distinguishing various processes. (**a**,**b**) Exciton population difference Δ*N*_1*s*,2*p*_ (red diamonds), Δ*N*_1*s*,3*p*_ (blue dots) as a function of time delay in a logarithmic scale at 8 K for 790 nm (550 μJ cm^−2^) and 399 nm (120 μJ cm^−2^) excitation, respectively. The detailed THz time scan with 50 fs resolution is shown together in **a**. The error bars of carrier densities indicate the uncertainty from theoretical fitting of the experimental results. (**c**) The time-dependent density *N*_*eh*_ of unbound carriers for two pumping conditions. (**d**) Time-dependent ratios of the population difference Δ*N*_1*s*,2*p*_/Δ*N*_1*s*,3*p*_ are shown in the inset, which allows to extract the cooling curves for two pumping conditions. (**e**) The photoinduced THz transmission under 790 nm and 550 μJ cm^−2^ pumping for various temperatures. A two-step rise, 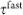
∼1.0±0.03 ps (grey) and 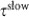
∼11.2±1.06 ps (cyan) is separated for the 8 K trace.

Access to both the 2*p* and higher-lying, 3*p*, dark bound states has been very scarce in materials^21^,^31^, which allows us to quantitatively analyse distribution functions and cooling curves of excitons in the perovskite system to corroborate these observations. The distinct spectral shapes in Fig. 3b,c allow us to faithfully extract the effective transition strength (see Supplementary Fig. 5 and Supplementary Note 4) and, thereby, densities of excitons (Fig. 4a,b) and unbound carriers (Fig. 4c). For the below-resonance excitation at 790 nm and 550 μJ cm^−2^, Fig. 4a presents the time-evolution of the extracted exciton population differences Δ*N*_1*s*,2*p*_=*N*_1*s*_−*N*_2*p*_ and Δ*N*_1*s*,3*p*_=*N*_1*s*_−*N*_3*p*_. They both show the same delayed buildup in time, which yields a nearly time-independent ratio 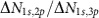
∼98% (red squares in the inset of Fig. 4d). This result indicates that the incoherent exciton population after fs photoexcitation is mostly distributed at the 1*s* state, that is, Δ*N*_1*s*,2*p*_≈Δ*N*_1*s*,3*p*_≈*N*_1*s*_. This is, again, in contrast to pump excitation of continuum states at 399 nm and 120 μJ cm^−2^, where the Δ*N*_1*s*,2*p*_ and Δ*N*_1*s*,3*p*_ now exhibit substantially longer rise times on 10s of ps time scale and display different temporal dependence (Fig. 4b) as compared to the 790 nm excitation (Fig. 4a). This results in a time-dependent population ratio varying by >10% over 60 ps (blue dots in the inset of Fig. 4d). This allows us to extract the cooling curve of the hot state as shown in Fig. 4d, that is, the thermalized, transient electronic temperature T* as a function of time (see Supplementary Note 4). T* is ∼270 K after the 399 nm excitation and gradually reaches lattice temperature of 8 K on a 10s of ps time scale (blue dots), while T* is already close to 8 K after the 790 nm excitation (red squares).

The distinct rise times of the relatively cold 1*s* exciton populations associated with the below-resonance pumping underpin a formation pathway different from the cooling of high-energy carriers. The time-dependent exciton density faithfully follows the detailed THz time scan with 50 fs resolution (black line, Fig. 4a) under 790 nm pumping, which displays a two-step rise with characteristic times 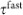
∼1.0±0.03 ps and 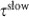
∼11.2±1.06 ps at 8 K (blue dots, Fig. 4e). We attribute this two-step exciton buildup to (1) the loss of exciton coherence and (2) the resonant scattering of 1*s* exciton with finite momentum distribution with discrete THz phonon modes *B*_1_–*B*_4_ in CH_3_NH_3_PbI_3_. Initially the formation of incoherent 1*s* exciton populations occurs on a timescale determined by the quantum dephasing time that characterizes the optical coherence-to-population conversion arising from multiple scattering contributions and disorder effects. The linewidths of the observed resonant internal THz transitions indicate optical polarization dephasing times of the order of 1 ps, which is consistent with the measured *τ*^fast^. Subsequently, the presence of discrete phonon states, with energies on the order of 2–8 meV comparable to the exciton kinetic energies, are unique, which make the 1*s* exciton populations with finite momenta relax to the *K*=0 state on a timescale of *τ*^slow^. This is much faster than conventional semiconductors. For example, the near-resonant photoexcitation in GaAs relaxes through acoustic phonon scattering which slows thermalization by orders of magnitude because of the absence of THz LO phonons^2^ (see Discussion and Supplementary Note 5). In addition, the delayed formation observed becomes faster with increasing temperature, for example, 8 and 100 K versus 160 K in Fig. 4e, consistent with thermally-induced dephasing and phonon scattering.

The density *N*_*eh*_ of unbound carriers following fs photoexcitations is shown in Fig. 4c. The *N*_*eh*_ exhibits a quasi-instantaneous, sub-ps generation for both continuum (white squares) and below-resonance (red squares) pumping, that is, the rise times exclusively in the photo-generated excitons (Fig. 4a). The efficient carrier generation is much faster than the exciton formation and independent of pump frequency. Intriguingly, the co-existence of both excitons and localized *e*-*h* carriers appears to be ‘universal' in the perovskites even at low temperature where *E*_1*s*_>*k*_B_*T*. This may be related to below-band-gap trapped states which separate *e* and *h* in real space and prevents them from coming close to each other within Bohr radius ∼5.2 nm to bind into excitons. The results of the Drude–Smith fitting, following Fig. 3a (dashed blue lines), yield a DC mobility of 23–69 cm^2^ V^−1^ s^−1^ for our sample.

## Discussion

Ultrafast intra-Rydberg–state transitions demonstrated here represent one of the most powerful methods to measure exciton formation pathways and high-frequency charge transport at the initial, highly non-equilibrium and non-thermal timescales following light pumping. The complete characterization of full response functions of excitons, charge carriers and phonons have not been possible in prior measurements in the perovskites. On the one hand, the results here show that the non-equilibrium emergent quantum states dynamically evolve with a complex co-existence of excitons, unbound charge carriers and phonons, for example, even at 10s of ps after photoexcitation as demonstrated in Fig. 3a. On the other hand, this allows us to directly reveal, previously inaccessible, multiple sharp quantum transitions and slow buildup of their correlated states under below resonance pumping. Particularly, the sub-ps resolved, transient exciton density in Fig. 4a clearly displays two-step formation pathways, while the unbound charge carriers show no such rise times (Fig. 4c). In addition, the transient temperatures of the excitonic reservoir are directly determined and already close to an initial lattice temperature after ultrafast photoexcitations, as shown in Fig. 4d. These facts unambiguously underpin a formation pathway different from the cooling of hot electronic and phonon reservoirs under the high energy, continuum pumping. Such unprecedented details of the non-equilibrium states are extremely difficult, if not impossible, to obtain from other measurements.

The existence of the distinct initial temporal regime under below-resonance pumping, characterized by a slow formation of the exciton populations, can be consistently explained by the loss of electronic coherence induced by laser light and the incoherent relaxation induced by the scattering of various discrete THz phonons. As it is well-established in prior work of exciton formation in GaAs^1^,^2^, there are two main processes that contribute to delayed exciton buildup when the pump excites below the exciton peak. First, the laser field couples to coherent *e*-*h* excitation amplitudes with zero centre-of-mass (CM) momentum when momentum is a good quantum number. In the case of strong disorder or phonon shakeup processes, a distribution of finite CM momentum excitons is excited. As a second step, scattering processes, mainly with phonons, transform such coherent *e*-*h* pair excitations into incoherent electron and hole populations with different momenta, or redistribute the photoexcited excitons among different CM states along the 1*s* parabola. Such populations resulting from exciton-phonon scattering grow as the *e*-*h* polarization dephases following laser excitation. Therefore, initially the buildup of low-energy 1*s* exciton populations occurs on a timescale determined by the optical polarization dephasing time, which arises from multiple scattering contributions and disorder effects, while later it is dominated by scattering with discrete or continuum phonons. The nearly ps formation time is supported by the linewidths of the observed THz responses, which is also largely consistent with a recent experiment showing *T*_2_ times of the same materials ∼600 fs at 10 K^35^. Second, following dephasing of the laser-driven exciton polarization, higher CM momentum states in the 1*s* exciton parabola become populated. Subsequently, these exciton populations relax towards the lowest energy states with small momenta by scattering with phonons. Unlike in GaAs and other inorganic semiconductors, here we observe discrete THz phonon states with energies on the order of 2–8 meV, that is, in the few THz range, as shown in Fig. 2b,d. Such phonon energies are comparable to the exciton CM kinetic energies. Excitons with large CM momentum can lose finite kinetic energy by a single scattering event with a discrete phonon, which absorbs the CM momentum. Optical phonons in GaAs are at much higher energy ∼36 meV. Here, the presence of discrete THz phonon states in the perovskite materials can lead to carrier and exciton relaxation on a timescale of few ps, much faster than scattering with acoustic phonons in the case of near–resonance pumping in GaAs ∼1 ns (ref. 2). Comparing between the perovskite materials here and GaAs provides a natural way to explain the similarities and differences in their exciton formation times. This also warrants future investigations to further explore other mechanisms also relevant of explaining these results.

Although our scheme and the samples chosen in this study are mostly relevant for revealing the fundamental ultrafast photo-conversion pathways and electronic coherence, we briefly discuss below about their potential impacts to the perovskite-based devices which operate mostly at room temperatures and in the form of thin films. First, the versatile THz spectroscopy tool shown here allows to measure the full response functions of both excitons and charge carriers under highly non-equilibrium conditions, which has not been possible in prior measurements in perovskite materials. This ultrafast characterization method is extremely relevant for understanding and potentially engineering high-speed electronic and optoelectronic devices. Therefore we expect that such a versatile tool may evolve into a benchmark characterization method for quantitative carrier/exciton management when applied to working devices. Second, the efficient carrier generation revealed here, much faster than the exciton formation even at low temperature, under resonant excitation and in crystal samples, attests the suitability of the perovskite materials for solar cells and photovoltaics. For example, the long exciton formation and cooling times, as compared to GaAs, should benefit the hot-state delocalization scenario for device operation as discussed in ref. 5. In addition, one should expect even more efficient photocarrier generation and quasi-instantaneous probability of energy migration in real device conditions because of the presence of many more defects/grain boundaries and elevated temperatures.

In summary, excitonic Rydberg states with intermediate binding energy, nearly ps dephasing time, and efficient carrier generation and THz phonon scattering in the perovskites are distinct from conventional semiconductors. The ultrafast, emergent quantum states excited here, being evolving on similar time scales as photocarrier generation and transport, provide clear implications for novel energy migration mechanism involving electronic coherence.

## Methods

### Sample preparation

Methylammonium iodide (CH_3_NH_3_I) and methylammonium lead (II) iodide (CH_3_NH_3_PbI_3_) were prepared by slightly modified literature procedures^7^. Briefly, hydroiodic acid (10 ml, 0.075 mol) was added to a solution of methylamine (24 ml, 0.192 mol) in ethanol (100 ml) at 0 °C while stirring, and stirring continued for 2 h. The solution was concentrated under vacuum, first in a rotary evaporator at 70 °C, and then under dynamic vacuum at 60 °C for 12 h. The remaining solid was recrystallized from ethanol. A solution of CH_3_NH_3_I (9.2 mg, 0.06 mmol) and PbI_2_ (9.6 mg, 0.02 mmol) in *γ*-butyrolactone (4 ml) was injected into toluene (15 ml) while stirring, and allowed to stand for 2 h at room temperature. The product was isolated by centrifugation (5 min at 4,500 r.p.m.) and washing with toluene (5 ml). We study a free-standing 550 μm thick perovskite-poly(methylmethacrylate) thin film that was made of embedding μm size, CH_3_NH_3_PbI_3_ crystals (Fig. 1b) in PMMA matrix. CH_3_NH_3_PbI_3_ (6 mg, 0.01 mmol) was dispersed in a solution of PMMA (0.13 g, 0.8 μmol) in toluene (3 ml), while sonicating and agitating until the mixture became homogeneous. A homogeneous solution of CH_3_NH_3_PbI_3_ and PMMA in toluene was prepared, cast into a mould and allowed to dry to optical quality films under ambient conditions.

### Ultrafast THz conductivity

To obtain the time-resolved, complex THz conductivity of the sample, Fig. 2a shows an example of the measured THz electric fields in time-domain, which are transmitted through a clear aperture (*E*_air_(*t*), grey) and sample (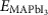
(*t*), black) in the absence of pump. It also shows the pump-induced change Δ
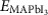
(*t*) (red) at a pump-probe delay Δ*t*=60 ps under 790 nm pumping and 550 μJ cm^−2^ fluence (pump #1, Fig. 1e). Note that there is negligible pump-induced change for a pure PMMA control sample Δ*E*_PMMA_(*t*) (blue). Specifically, the static and photoexcited complex transmission coefficients with the spectral amplitude and phase information are obtained from the data, from which, together with the application of the effective medium approximation (EMA) (See Supplementary Note 3), the static complex dielectric function 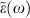 and its pump-induced change 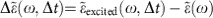 are numerically retrieved. The corresponding complex conductivities are calculated by the equation 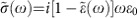. The real part of the conductivity Δ*σ*_1_(*ω*) and of the dielectric function Δ*ɛ*_1_(*ω*) are presented in the study, which measures the dissipative and inductive parts of response function. Please note that time resolution taken as the THz time scan in Fig. 4a (black line) is limited by gate pulse duration of 50 fs instead of THz pulse duration.

### Theory and fitting

The experimentally-determined, sub-ps THz response functions have been fitted by a model that assumes co-existence of excitons, phonons and *e*-*h* plasma (see Supplementary Note 4). Specifically, the simultaneously-obtained, frequency-dependent complex conductivity on a sub-ps time scale allows us to quantitatively monitor the dynamic evolution of excitons and unbound *e*–*h* plasma, which have distinctly different spectral features in pump-induced changes Δ*σ*_1_(*ω*) and Δ*ɛ*_1_(*ω*). We construct a THz line-shape model consisting of three components: the THz dielectric function of resonant excitonic absorptions (first and second terms), resonant phonon bleaching (third term) plus a Drude–Smith (DS) component of unbound *e*-*h* plasma (fourth term), as shown in equation (1). The first two terms accounts exactly the internal quantum transition of the excitonic Rydberg states 1*s*→*np* (*n*=2,3)


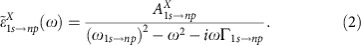


These account for the correlated, dissipative and inductive features, exclusively below *T*_*S*_, from the internal, resonant quantum transitions between the Rydberg states. Here 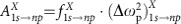, where *f* is the oscillator strength, 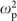 is the plasma frequency *N*_*X*_*e*^2^/(*ɛ*_0_*μ*), *N*_*X*_ is the exciton density, *e*, *μ* and *ɛ*_0_ are the electron charge, exciton effective mass, and vacuum permittivity, respectively. *ω*_1*s*→*np*_ is the excitonic 1*s*→*np* transition resonant frequency and Γ is the broadening. Therefore, the 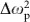 measures the population difference between the two Rydberg states involved in the transitions (Fig. 1a), that is, Δ*N*_1*s*,*np*_=*N*_1*s*_−*N*_*np*_ (ref. 22). Other intra-excitonic quantum transitions associated with higher-lying *np* bound states (*n*≥4) and band continuum make negligible contributions to Δ*σ*_1_ up to 14 meV. In the fitting, oscillator strength *f* is calculated using equation (5). *e* and *ɛ*_0_ are constant. Exciton effective mass μ=0.104*m*_e_ is taken from ref. 15. And *ω*_1*s*→*np*_, Γ_1*s*→*np*_, and Δ*N*_1*s*,*np*_ are varied to match the experimentally obtained excitonic resonant frequencies, bandwidths, and amplitudes centred at *A*_1_∼10.1 meV and *A*_2_∼12.1 meV, respectively.

Next, the third term in equation 1 describes the THz dielectric function from photoinduced phonon bleaching modes of the CH_3_NH_3_PbI_3_ crystals (*B*_*j*_, *j*=1–4) and PMMA matrix (*P*_*k*_, *k*=1–2), which is given by


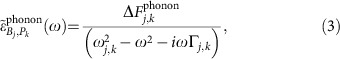


where 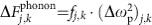, similar to parameter definition of equation (2), and additionally, *ω*_*j*,*k*_ and Γ_*j*,*k*_ are the phonon resonant frequencies and broadenings, respectively. Note besides the phonon modes in the sample, marked as B_*j*_ in Fig. 2d, two phonon modes *P*_1_∼10 meV (2.4 THz) and *P*_2_∼26.7 meV meV (6.5 THz) are added from the PMMA matrix as confirmed by a separate THz time-domain (red) and FTIR (black) measurements, as shown in Supplementary Fig. 4. In the fitting, 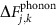, *ω*_*j*,*k*_, and Γ_*j*,*k*_ are varied to match the experimentally obtained phonon strengths, resonant frequencies, and bandwidths for B_*j*_ and P_*k*_, respectively. Note the high frequency mode at 26.7 meV (6.5 THz) only contributes as a ‘featureless' background in Δ*ɛ*_1_(*ω*) since the measured spectra region is below 3.5 THz (14.5 meV) (Fig. 2b,c).

The last term in equation (1) describes the non-resonant component from the Drude-Smith term


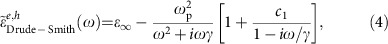


where the plasma frequency 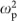
=*N*_*eh*_*e*^2^/(*ɛ*_0_*μ*_*e*_), *N*_*eh*_ is unbound free charge carrier density. And *e*, *ɛ*_0_, and *ɛ*_∞_ are the electron charge, vacuum and background permittivity. *γ* is the electron broadening. The electron effective mass *μ*_*e*_=0.19*m*_e_ is taken from ref. 36. When the backscattering coefficient *c*_1_=0, the standard Drude model under the free electron approximation is recovered. When *c*_1_=−1, the DC conductivity is vanishing which manifests as backscattering of carriers. The photoinduced conductivity Δ*σ*_1_(*ω*) is suppressed (Fig. 2b) and Δ*ɛ*_1_(*ω*) increases rapidly (Fig. 2c) as *ω* → 0, in contrast to the conventional Drude characteristics. Instead, these are consistent with Drude–Smith model predictions for disorder-/backscattering-induced transport of free electrons which have been extensively studied in carrier transport in nanocrystals and dye-sensitized TiO_2_ as shown in ref. 32. In the fitting, *e*, *ɛ*_0_, *ɛ*_∞_ and *μ*_*e*_ are constant. *N*_*eh*_, *γ* and *c*_1_ are varied to match the experimentally obtained Drude–Smith amplitude, broadening and depression of the DC conductivity, respectively. Drude–Smith model can be used to consistently fit the observed Δ*σ*_1_(*ω*) and Δ*ɛ*_1_(*ω*) with parameter *c*_1_ in the range of −0.96 to −1 for all temperatures from 8 to 295 K, which yields a DC mobility of 23–69 cm^2^ V^−1^ s^−1^ as *ω*→0 in our sample. In addition, the observed, broad THz background line shapes can be also fitted by the Plasmon model which is identical to the Drude-Smith model in our case for c_1_=−1 and c_*p*_=0 with *p*>1. The calculated carrier densities from these 2 models are the same, except that the scattering rate of Plasmon model is twice of that of Drude-Smith model. Using Plasmon model will not change our conclusion of intra-exciton transitions of the paper. Given the fact that Plasmon resonances are highly shape dependent, random shapes seen in our samples make it hard to be applicable.

### THz lineshape analysis

For exciton dynamics, our THz lineshape analysis yields the time-evolution of exciton population and carrier density shown in Fig. 4a–c. In the resonant coherent excitation regime, the intrinsic microscopic pathway for the buildup of exciton population is shown to originate from the dephasing of electronic coherence and scattering with discrete phonons. The polarization-to-population conversion time is shown to be on ∼1 ps time scales. In the largely incoherent regime with *e*-*h* continuum excitation, cooling of the hot population can be directly accessed based on the fitted, time-evolution of exciton population difference *N*_1*s*_−*N*_2*p*_ and *N*_1*s*_−*N*_3*p*_ with a given distribution function. The oscillator strength used can be determined theoretically based on the hydrogen model. For 1*s*→*np* transition, they can be expressed as


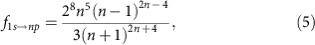


which gives *f*_1*s*→2*p*_=0.416 and *f*_1*s*→3*p*_=0.079. Since excitons are composite bosons, with repulsive interaction to the second order, formed in a fermion many-body systems, one may not simply expect a pure Bose–Einstein or rigorous Fermi–Dirac distribution functions of hot excitons. Assuming that quasi-equilibrium has been reached at early stage and the Fermi-Dirac distribution, we further extract the effective temperature T* evolution of the excitons using the relation


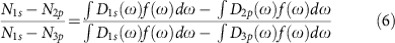


where the density of states of the *ns*/*p* excitons in the three-dimensional space is given by


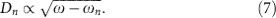


Given the experimentally-extracted ratio Δ*N*_1*s*,2*p*_/Δ*N*_1*s*,3*p*_ (inset, Fig. 4d), we calculate the effective temperature T* using the above relations. Figure 4d shows that excitons stimulated by the 399 nm pulses are initially at quite high temperature, and gradually cool down on 10 s of ps time scale. Meanwhile, the excitons generated by the 790 nm pulses are already very cool, close to the lattice temperature at the early stage. Finally, Fig. 4c shows that the photoexcitation quasi-instantaneously convert into mobile carriers, despite the absence of longer range transport hindered by disorder and/or crystal boundary as seen in the suppressed Δ*σ*_1_(*ω*) as *ω*→0.

### Data availability

The datasets generated during and/or analysed during the current study are available from the corresponding author on reasonable request.

## Additional information

**How to cite this article:** Luo, L. *et al*. Ultrafast terahertz snapshots of excitonic Rydberg states and electronic coherence in an organometal halide perovskite. *Nat. Commun.* **8**, 15565 doi: 10.1038/ncomms15565 (2017).

**Publisher's note**: Springer Nature remains neutral with regard to jurisdictional claims in published maps and institutional affiliations.

## Supplementary Material

Supplementary Information Supplementary Figures, Supplementary Notes and Supplementary References
