# Supplementary material for: Ultrafast terahertz snapshots of excitonic Rydberg states and electronic coherence in an organometal halide perovskite
Source: Nat Commun. 2017 Jun 1;8:15565. doi: 10.1038/ncomms15565 (PMC5461501; doi:10.1038/ncomms15565)
Supplement: Supplementary Information — Supplementary Figures, Supplementary Notes and Supplementary References [file ncomms15565-s1.pdf]

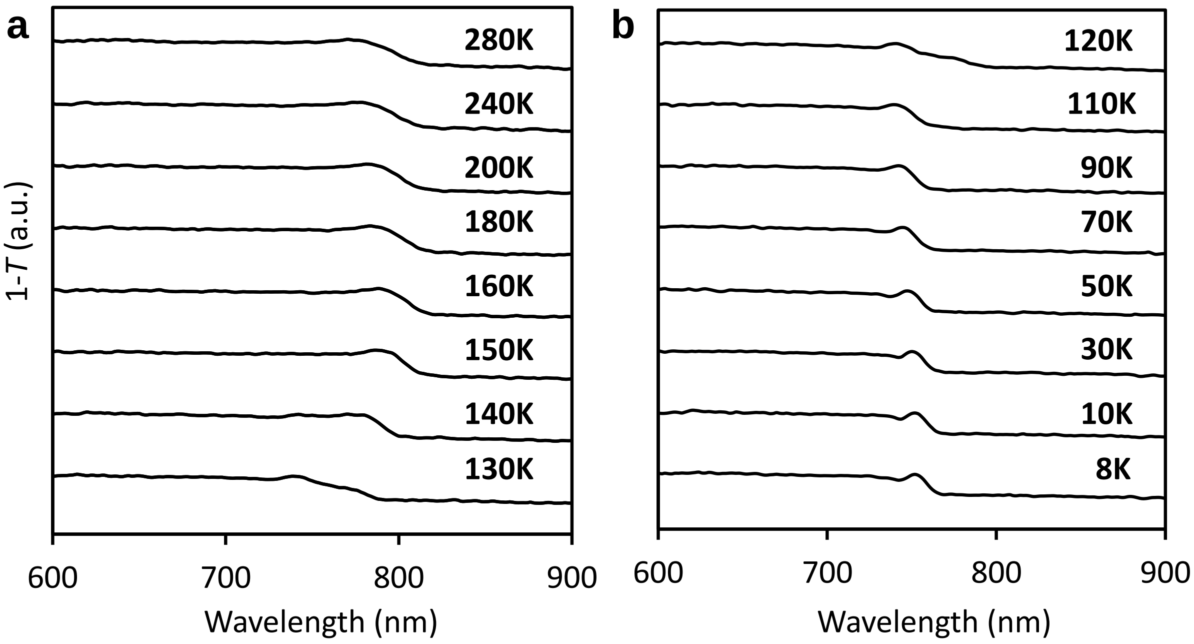

Supplementary Figure 1. Temperature dependent transmission measurement. The transmission spectra of the $CH_3NH_3PbI_3$/PMMA film plotted as 1-T from 8 K to 280 K.

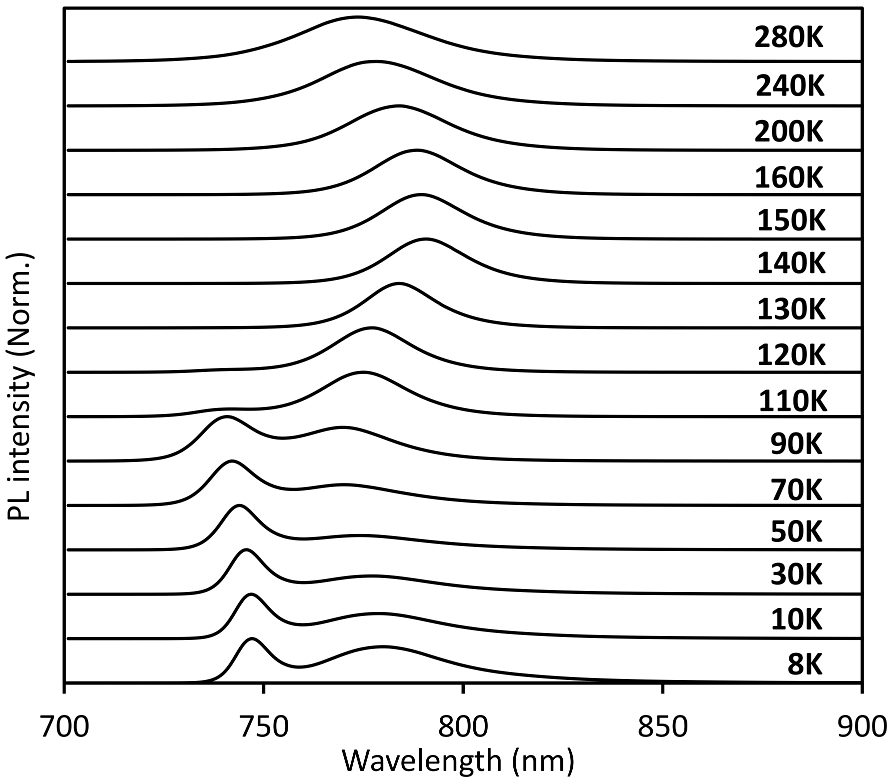

Supplementary Figure 2. Temperature dependent photoluminescence measurement. The photoluminescence spectra of the $CH_3NH_3PbI_3$/PMMA film measured from 8 K to 280 K. The sample is excited by 632.8 nm He-Ne laser.

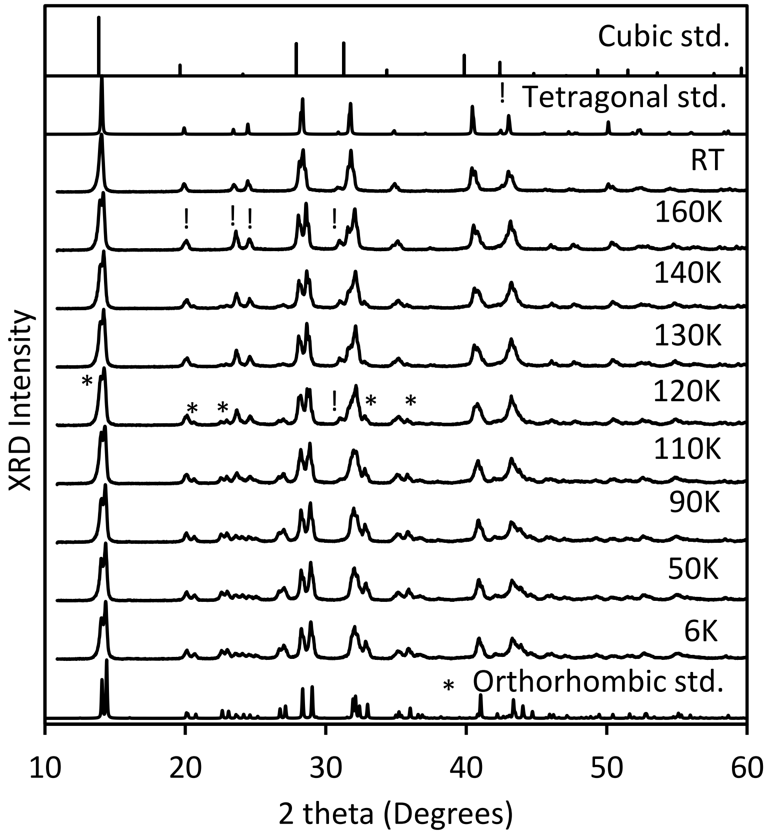

Supplementary Figure 3. Temperature dependent XRD measurement. The XRD measurement of the sample from 6 K to room temperature (∼295 K). The standard cubic, tetragonal, and orthorhombic XRD curves are plotted as a reference to compare with experimental results.

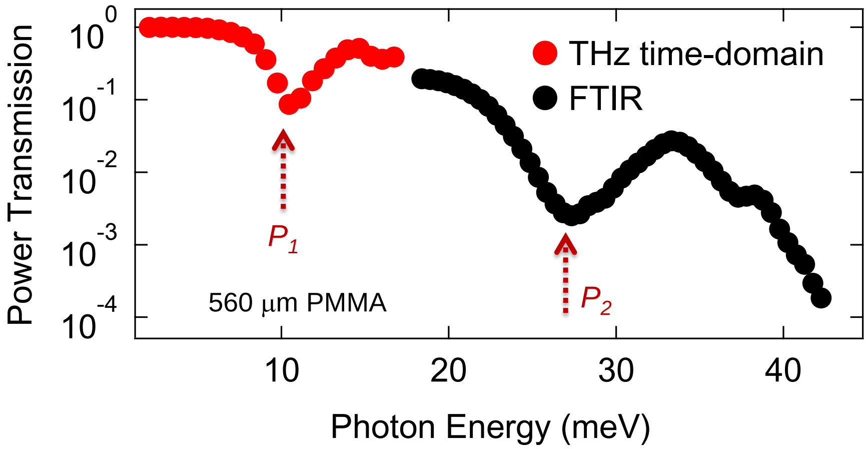

Supplementary Figure 4. Transmission measurement of a pure PMMA substrate. The transmission spectra of a 560 μm thick pure PMMA is measured by THz and FTIR spectrometers, respectively. Two phonon modes around 10 meV and 26.7 meV are observed, which contribute as a featureless background in pump-induced THz conductivity.

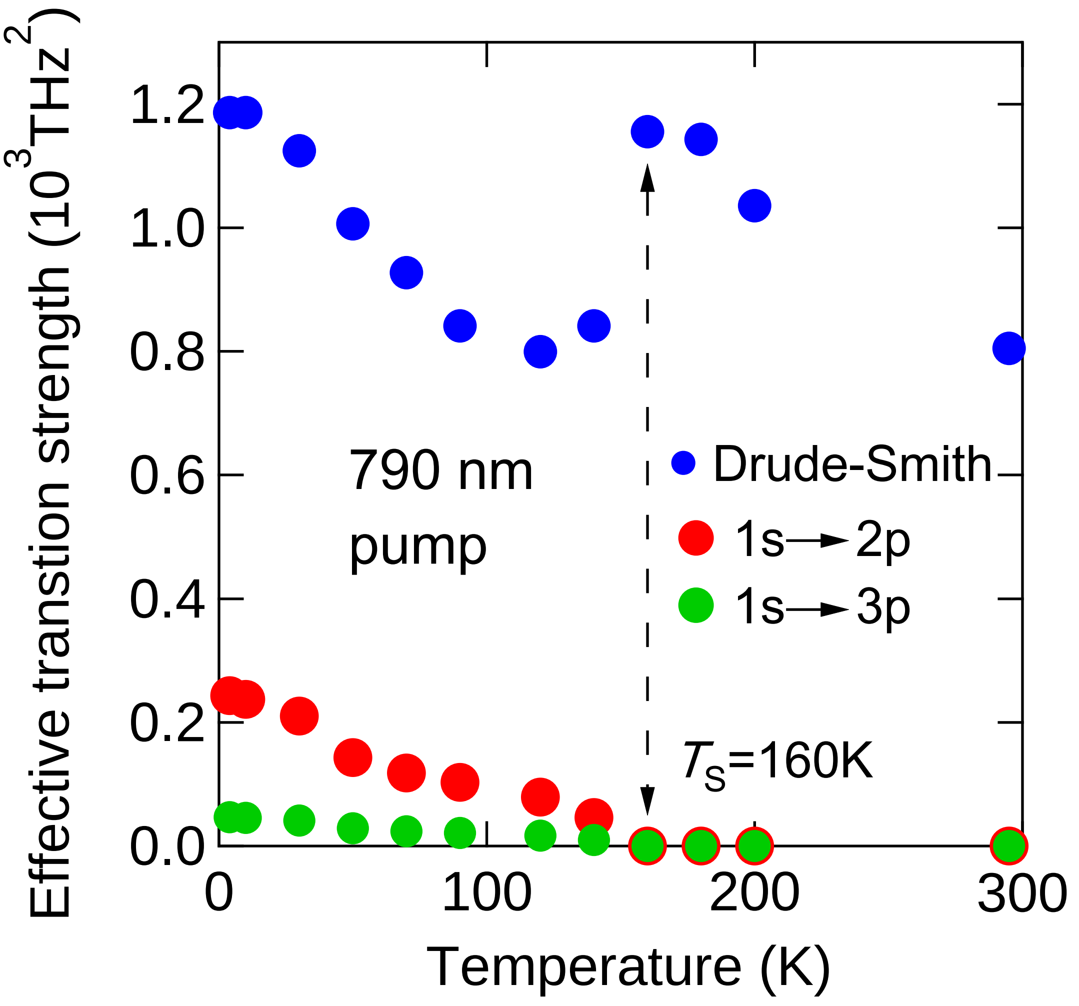

Supplementary Figure 5. Effective transition strengths at various temperatures. The effective transition strengths of the intra-excitonic transitions 1s→2p $A^X_{1s\to 2p}$ (red dots), 1s→3p $A^X_{1s\to 3p}$ (green dots), and unbound carriers A^{eh}_{DS} (blue dots) are presented as ω_{p}^2. This behavior is fully consistent with the perovskite phase transition and it is highly unlikely to assign these features to PMMA.

In the Supplementary Information, we present more details on experimental techniques, samples, extra results, data analysis and theoretical modeling.

1 Supplementary Note 1: Introduction

Organometal halide perovskite materials have recently emerged as one of the most exciting photovoltaic and opto-electronic materials due in part to their unique properties such as strong visible-light absorption, high charge mobility, long charge-carrier lifetime and diffusion lengths. The determination of exciton binding energy and conversion pathways remains challenging in the hybrid organic-inorganic perovskites. This key missing information serves as a foundation to design efficient photoconversion schemes and device architectures based on these materials. Since the exciton formation also intimately relates to the loss of electronic coherence and conversion between Coulomb-bound and unbound e-h pairs, such investigation in organometal halide perovskite provides rare opportunities of learning more about these fundamental quantum processes. The lack of quantitative, ultrafast quasi-particle spectroscopy tools and the inability to elucidate initial quantum dynamics in the hybrid perovskites seriously limit both the thorough understanding of their photoconversion mechanism, and our perspectives of developing coherent quantum devices.

Here, we present a clear-cut picture, with an unprecedented level of details, of the THz quantum and thermal transport in perovskite materials. We reveal the ps coherent dynamics, excitonic Rydberg states with intermediate binding energy, and discrete THz phonon scattering that are distinct from conventional photovoltaic systems. We show resonant, internal quantum transitions of excitons and elucidate the initial coherent and cooling dynamics of exciton formation in a model hybrid perovskite $CH_3NH_3PbI_3$ ($MAPbI_3$) by generating and detecting

electronic coherence, excitons and carrier plasma using direct, ultrafast THz quasi-particle spectroscopy. Ultrafast THz spectra provide evidence for two strong photoinduced resonant absorption peaks centered at $\sim$10.1 meV and 12.1 meV, which manifest the observation of the internal, excitonic $1s \rightarrow 2p$ and $1s \rightarrow 3p$ transitions. These precisely yield the binding energy 13.5 meV and appear exclusively below the tetragonal-to-orthorhombic structural phase transition at T_S=160 K. We further elucidate the initial coherent and cooling dynamics of excitons and carrier plasma. Our results provide the first insights into the coherence-to-population scattering process following near-resonant, fs near-IR photoexcitation, which forms "cold" exciton population with the vanishing of electronic coherence on $\sim$1 ps time scales. This initial coherent process is separated in time from the carrier-discrete THz phonon scattering $\sim$10 ps. In contrast, off-resonant, fs UV photoexcitation provides access to the hot electronic state, whose distributions relax to the excitonic ground state on $\sim$10s of ps time scale by the energy-loss process limited by continuum acoustic phonon scattering. Our analysis of the time-resolved THz spectra, as detailed below, quantitatively determines the coherence- and cooling-limited formation times and cooling curves of the hot state. Furthermore, the measured complex conductivity spectra at ultrafast time scale reveal a robust and concurrent responses of both the excitons and mobile e-h plasma, though their bulk transport is hindered by disorder-induced localization and backscattering. Our results establish a framework to understand the fundamental conduction mechanism and to benefit the development of photoconversion devices in hybrid perovskite materials.

2 Supplementary Note 2: Sample preparation and basic characterizations

Lead (II) iodide (PbI_2, 99%), methylamine solution (33 wt% in absolute ethanol) and γ-butyrolactone ($\geq$99%) were purchased from Sigma-Aldrich; hydroiodic acid (ACS, 55-58%) from Alfa-Aesar;

poly(methylmethacrylate) (P2675-MMA) from Polymer Source Inc.; and toluene (99.9%) from Fisher. All chemicals were used as received.

Methylammonium iodide (CH_3NH_3I) and methylammonium lead (II) iodide ($CH_3NH_3PbI_3$) were prepared by slightly modified literature procedures [7]. Briefly, hydroiodic acid (10 mL, 0.075 mol) was added to a solution of methylamine (24 mL, 0.192 mol) in ethanol (100 mL) at 0 °C while stirring, and stirring continued for 2 h. The solution was concentrated under vacuum, first in a rotary evaporator at 70 °C, and then under dynamic vacuum at 60 °C for 12 h. The remaining solid was recrystallized from ethanol. A solution of CH_3NH_3I (9.2 mg, 0.06 mmol) and PbI_2 (9.6 mg, 0.02 mmol) in γ-butyrolactone (4 mL) was injected into toluene (15 mL) while stirring, and allowed to stand for 2 h at room temperature. The product was isolated by centrifugation (5 min at 4500 rpm) and washing with toluene (5 mL).

We study a free-standing 550 μm thick perovskite-poly(methylmethacrylate) thin film ($CH_3NH_3PbI_3$/PMMA) that was made of embedding μm size $CH_3NH_3PbI_3$ crystals (Fig. 1b in the main text) in PMMA matrix. $CH_3NH_3PbI_3$ (6 mg, 0.01 mmol) was dispersed in a solution of PMMA (0.13 g, 0.8 μmol) in toluene (3 mL), while sonicating and agitating until the mixture became homogeneous. A homogeneous solution of $CH_3NH_3PbI_3$ and PMMA in toluene was prepared, cast into a mold and allowed to dry to optical quality films under ambient conditions, as shown in SEM image of the cross-section of $CH_3NH_3PbI_3$/PMMA film. (Fig. 1c). Room temperature X-ray diffraction (XRD) measurements of $CH_3NH_3PbI_3$/PMMA film and pure $CH_3NH_3PbI_3$ powder corroborate the inclusion of the perovskites into PMMA matrix, as shown in Fig. 1d of the main text.

To fully characterize the low temperature phase transition behavior in the samples, we performed low temperature transmission (Supplementary Figure 1), photoluminescence (PL,

Supplementary Figure 2) and X-ray diffraction (XRD) measurements of $CH_3NH_3PbI_3$ sample down to 6 K (Supplementary Figure 3). We paid special attentions for the behaviors across the tetragonal-to-orthorhombic structural phase transition at T_S=160 K. The temperature dependent XRD measurements were performed using the $CH_3NH_3PbI_3$ powder (particles in μm size) at temperatures ranging from 6 K to room temperature (295 K). The powder was mixed in the copper sample holder with GE varnish (commercial product) using toluene as a solvent and let dry for 24 hours at ambient conditions; then the solidified material was polished to create a flat surface suitable for the powder XRD data acquisition. The data were collected on a Rigaku TTRAX system with a rotating anode generating Mo Kα radiation. The diffractometer was equipped with a continuous flow ^{4}He cryostat controlling the temperature of a sample. The range of measured Bragg angles was from 10 to 60 degrees of 2θ; the time of one temperature measurement was near two hours.

These observations shown in Supplementary Figures 1-3 are qualitatively consistent with the prior studies of high-quality perovskite materials [12,13,18]. Specifically, these data clearly indicate that the room-temperature tetragonal phase of the system is lowered to an orthorhombic one starting at the temperature T_S ~160 K. The new structure fully sets in ~120 K as seen in the low temperature XRD data (Supplementary Figure 3), which correlates with a complex spectral shaping in the absorption (Supplementary Figure 1) and PL (Supplementary Figure 2) traces. Outside this transition region, both absorption and PL data exhibit a simpler, redshift of band edge by decreasing the temperature, i.e., the 'Varshni' trend in lead composite semiconductors.

The spectra of the 790 nm (pump #1) and 399 nm (pump #2) pump beams are also plotted in Fig. 1e in order to compare them with the absorption spectra. This way, fundamentally different initial excitation conditions can be selectively generated by tuning the pump photon

energy, e.g., “cold” excitons and hot e-h plasma can be generated via resonant excitation close to $1s$–exciton or off-resonant into interband continuum. These enable the unique study of the distinct initial conditions and their ultrafast evolutions, as shown in Figs. 3b-3c in the main text.

Our samples containing individual single perovskite crystals free from interactions are much more preferable to extract the true fundamental physics of the general perovskite materials, e.g., ultrafast exciton formation pathways and associated coherent transient effects. Particularly, we aim to underpin the similarities and differences of the perovskite materials compared to conventional optoelectronic semiconductor materials such as GaAs, which is also in a single crystal form, in order to further establish the new organic-inorganic perovskite systems as new revolutionary materials for light-harvesting and electron-transporting applications. In contrast to our samples, it is well established that electronic coherence and charge transport of thin film samples are dominated by the microstructural features such as grains and grain boundaries (10-1000 nm), which make it extremely difficult to study the universal and intrinsic physics of the materials by using such samples. Nevertheless, we think that the sample comparison should be performed in the future work.

3 Supplementary Note 3: Experimental details

We perform optical-pump and THz-probe spectroscopy, which is driven by a 1 kHz Ti:Sapphire regenerative amplifier with 790 nm central wavelength and 40 fs pulse duration. One part of the output is used to pump the sample either directly at the fundamental wavelength of 790 nm or at 399 nm after going through a β-barium-borate (BBO) crystal. The pump spectra are shown in Fig. 1e together with the linear absorption spectra of the $CH_3NH_3PbI_3$/PMMA film. The other part of the output is used to generate and detect phase-locked THz fields in time-domain

via optical rectification and electro-optic sampling of two 0.2 mm thick ZnTe crystals, respectively. This increases by an order of magnitude of the signal-to-noise ratio at the important, high frequency regions in the range of 10-15 meV comparing to 1 mm thick ZnTe emitter/detector. The THz electrical fields exhibit broadband spectral width from 2–15 meV used as a probe. We achieve a dynamic range of ~72 dB at the peak of THz spectrum, ~60dB at the $1s\rightarrow 2p$ and ~50dB at $1s\rightarrow 3p$ transitions. Two 2.5 mm diameter copper apertures are used, one of which is placed directly in front of the sample to ensure uniform photoexcitation, the other one of which is used as a reference to measure THz free propagation. THz section of the setup is enclosed in a dry air purge box in order to get rid of the THz absorption by water vapor. More details of the THz setup can be found in Ref [31]. To obtain time-resolved complex THz conductivity of the sample, we record the THz electrical fields in time-domain transmitted through a clear aperture $E_{\text{air}}(t)$, static sample $E_{\text{MAPbI}_3}(t)$, and its pump-induced change $\Delta E_{\text{MAPbI}_3}(t)$ at a fixed pump-probe delay Δt (Fig. 2a). Note there is negligible pump-induced change for a pure PMMA control sample $\Delta E_{\text{PMMA}}(t)$. The static and photoexcited complex transmission coefficients with the spectral amplitude and phase information are obtained from the data. Through the fast Fourier transformation and Fresnel equation, the THz dielectric responses can be obtained for both static and photoexcited samples. For photoexcited sample, the pump-induced refractive index change is assumed to have an exponential decay within the sample, i.e., $n(x) = n_0 + \Delta n\cdot\exp(-x/d)$, where n_0 is the averaged static refractive index obtained from static measurement including contributions from both perovskite crystals and PMMA matrix based on their ratio, Δn is the pump-induced refractive index change on the sample surface, d is the pump effective penetration depth obtained from absorption measurement by considering the sample as a homogeneous medium, and x is the distance that the light travels in the sample with x=0 at the entrance surface and x=L at the exit surface. L is the sample thickness. The dielectric function extracted this way is actually the averaged effective dielectric function consisting of contributions from both

perovskite crystals and PMMA matrix, e.g., $\widetilde{\varepsilon}^{\text{effective}}(\omega)$. Then, effective medium approximation (EMA) is applied in order to calculate the perovskite contribution $\widetilde{\varepsilon}^{\text{perovskite}}(\omega)$ from the averaged effective dielectric function. To do so, the space filling ratio (FR) of the sample $\sim 1.2\%$, calculated from the SEM images, is required, which leads to a simple relation of $\widetilde{\varepsilon}^{\text{effective}}(\omega) \approx \text{FR}\cdot\widetilde{\varepsilon}^{\text{perovskite}}(\omega)$ for FR much smaller than 1 as shown in ref[31]. For simplicity, we simply write $\widetilde{\varepsilon}^{\text{perovskite}}(\omega)$ as $\widetilde{\varepsilon}(\omega)$ throughout the paper. Therefore, the static complex dielectric function $\widetilde{\varepsilon}(\omega)$ and its pump-induced change $\Delta\widetilde{\varepsilon}(\omega, \Delta t) = \widetilde{\varepsilon}_{\text{excited}}(\omega, \Delta t) - \widetilde{\varepsilon}(\omega)$ are numerically retrieved. The corresponding complex conductivities are calculated by the equation $\widetilde{\sigma}(\omega) = i[1 - \widetilde{\varepsilon}(\omega)]\omega\varepsilon_0$. The real part of the conductivity $\Delta\sigma_1(\omega)$ and of the dielectric function $\Delta\varepsilon_1(\omega)$ are presented in the study, which measures the dissipative and inductive parts of response function. Applied to the current study, this allows to simultaneously obtain frequency-dependent complex conductivity associated with the dissipative and inductive response of excitons and unbound e–h carriers on a sub-ps time scale [31].

Our results reproduce the main features that are fully consistent with the prior studies. For example, our temperature-dependent transient THz spectra of $\Delta\sigma_1(\omega)$ and $\Delta\varepsilon_1(\omega)$ after 790 nm excitation and at pump-probe delay Δt=60 ps (Figs. 2b and 2c) clearly reveal four phonon bleaching modes B_j (j=1-4) whose frequency and temperature dependence are consistent with prior observations [24,25]. Most intriguingly, two photoinduced absorption oscillators centered at $\sim$10.1 meV and 12.1 meV can be attributed to intra-excitonic transitions of 1s$\rightarrow$2p and 1s$\rightarrow$3p of the perovskite Rydberg states. A complete temperature-dependent THz conductivities $\Delta\sigma_1(\omega)$ from 8 K to 295 K, as shown in Fig. 2d, clearly show that the two internal excitonic transitions exhibit maximum amplitude at 8 K, which gradually decrease and finally vanish by raising the lattice temperature above the structural phase transition.

As shown in Fig. 4a (main text), the time evolution of the photo-generated exciton density faithfully follows THz time scan (black line) with 50 fs resolution, which display a two-step formation pathways with characteristic times much slower than pump pulse duration of 50 fs. Please note that it has been well-established that time resolution taken at such a condition is limited by gate pulse duration of 50 fs instead of THz probe pulse. These formation times are distinctly different from other conventional photovoltaic materials.

4 Supplementary Note 4: Theory and fitting results

The simultaneously-obtained, frequency-dependent complex conductivity on a sub-ps time scale allows us to quantitatively monitor the dynamic evolution of excitons and unbound e–h plasma, which have distinctly different spectral features in pump-induced changes $\Delta\sigma_1(\omega)$ and $\Delta\varepsilon_1(\omega)$. We construct a THz line-shape model consisting of three components: the THz dielectric function of resonant excitonic *absorptions* (1st and 2nd terms), resonant phonon *bleaching* (3rd term) plus a Drude-Smith (DS) component of unbound e-h plasma (4th term):

$$\widetilde{\varepsilon}(\omega) = \widetilde{\varepsilon}^X_{1s\to 2p}(\omega) + \widetilde{\varepsilon}^X_{1s\to 3p}(\omega) + \sum_{j,k} \widetilde{\varepsilon}^{\text{phonon}}_{B_j,P_k}(\omega) + \widetilde{\varepsilon}^{e,h}_{\text{Drude-Smith}}(\omega). \quad (1)$$

The first two terms accounts exactly the internal quantum transition of the excitonic Rydberg states $1s$→np (n=2,3)

$$\widetilde{\varepsilon}^X_{1s\to \text{n}p}(\omega) = \frac{A^X_{1s\to \text{n}p}}{(\omega_{1s\to \text{n}p})^2 - \omega^2 - i\omega\Gamma_{1s\to \text{n}p}}. \quad (2)$$

These account for the correlated, dissipative and inductive features, exclusively below T_S, from the internal, resonant quantum transitions between the Rydberg states. Here $A^X_{1s\to \text{n}p} = f^X_{1s\to \text{n}p} \cdot (\triangle\omega_\text{p}^2)^X_{1s\to \text{n}p}$, where f is the oscillator strength, ω_p^2 is the plasma frequency $N_X e^2/(\varepsilon_0 \mu)$, N_X is the exciton density, e, μ, and ε_0 are the electron charge, exciton effective mass, and vacuum permittivity, respectively. $\omega_{1s\to \text{n}p}$ is the excitonic $1s \to \text{n}p$ transition resonant frequency and

Γ is the broadening. Therefore, the $\triangle\omega_{\rm p}^2$ measures the population difference between the two Rydberg states involved in the transitions (Fig. 1a), i.e., $\Delta N_{1s,\mathrm{n}p} = N_{1s} - N_{\mathrm{n}p}$ [22]. Other intra-excitonic quantum transitions associated with higher-lying np bound states (n≥4) and band continuum make negligible contributions to $\Delta\sigma_1$ up to 14 meV. In the fitting, oscillator strength f is calculated using Eq. 5 of the Supplementary Note 4. e and ε_0 are constant. Exciton effective mass μ=0.104$m_{\rm e}$ is taken from Ref[15]. And $\omega_{1s\to\mathrm{n}p}$, $\Gamma_{1s\to\mathrm{n}p}$, and $\Delta N_{1s,\mathrm{n}p}$ are varied to match the experimentally obtained excitonic resonant frequencies, bandwidths, and amplitudes centered at A_1 ~10.1 meV and A_1 ~12.1 meV, respectively.

Next, the third term in Eq. 1 describes the THz dielectric function from photoinduced phonon bleaching modes of the $CH_3NH_3PbI_3$ crystals (B_j, j=1-4) and PMMA matrix (P_k, k=1-2), which is given by

$$\widetilde{\varepsilon}^{\rm phonon}_{B_j,P_k}(\omega) = \frac{\Delta F^{\rm phonon}_{j,k}}{(\omega_{j,k}^2 - \omega^2 - i\omega\Gamma_{j,k})}, \tag{3}$$

where $\Delta F^{\rm phonon}_{j,k} = f_{j,k} \cdot (\triangle\omega_{\rm p}^2)_{j,k}$, similar to parameter definition of Eq. 2, and additionally, $\omega_{j,k}$ and $\Gamma_{j,k}$ are the phonon resonant frequencies and broadenings, respectively. Note besides the phonon modes in the sample, marked as B_j in Fig. 2d, two phonon modes P_1 ~10 meV (2.4 THz) and P_2 ~ 26.7 meV (6.5 THz) are added from the PMMA matrix as confirmed by a separate THz time-domain (red) and FTIR (black) measurements, as shown in Supplementary Figure 4. In the fitting, $\Delta F^{\rm phonon}_{j,k}$, $\omega_{j,k}$, and $\Gamma_{j,k}$ are varied to match the experimentally obtained phonon strengths, resonant frequencies, and bandwidths for B_j and P_k, respectively. Note the high frequency mode at 26.7 meV (6.5 THz) *only* contributes as a "featureless" background in $\Delta\varepsilon_1(\omega)$ since the measured spectra region is below 3.5 THz (14.5 meV) (Figs. 2b and 2c).

The last term in Eq. 1 describes the non-resonant component from the Drude-Smith term

$$\widetilde{\varepsilon}^{e,h}_{\text{Drude-Smith}}(\omega) = \varepsilon_\infty - \frac{\omega_{\text{p}}^2}{\omega^2 + i\omega\gamma}[1 + \frac{c_1}{1 - i\omega/\gamma}], \tag{4}$$

where the plasma frequency $\omega_{\text{p}}^2 = N_{eh}e^2/(\varepsilon_0\mu_e)$, N_{eh} is unbound free charge carrier density. And e, ε_0, and ε_∞ are the electron charge, vacuum and background permittivity. γ is the electron broadening. The electron effective mass μ_e=0.19m_{e} is taken from Ref[36]. When the backscattering coefficient c_1=0, the standard Drude model under the free electron approximation is recovered. When c_1=-1, the DC conductivity is vanishing which manifests as backscattering of carriers. The photoinduced conductivity $\Delta\sigma_1(\omega)$ is suppressed (Fig. 2b) and $\Delta\varepsilon_1(\omega)$ increases rapidly (Fig. 2c) as $\omega \to 0$, in contrast to the conventional Drude characteristics. Instead, these are consistent with Drude-Smith model predictions for disorder-/backscattering-induced transport of free electrons which have been extensively studied in carrier transport in nanocrystals and dye-sensitized TiO_2 as shown in REf [32]. In the fitting, e, ε_0, ε_∞, and μ_e are constant. N_{eh}, γ, and c_1 are varied to match the experimentally obtained Drude-Smith amplitude, broadening, and depression of the DC conductivity, respectively. Drude-Smith model can be used to consistently fit the observed $\Delta\sigma_1(\omega)$ and $\Delta\varepsilon_1(\omega)$ with parameter c_1 in the range of -0.96 to -1 for all temperatures from 8 to 295 K, which yields a DC mobility of 23–69 cm^2/(V·s) as $\omega \to 0$ in our sample. One can formally understand the localization of mobile carriers in the framework of Anderson localization, which is well established in early studies of the heavily doped conducting polymers, e.g., in Ref [37].

Moreover, Figs. 3a-3c in the main text highlight the responses exclusively from photogenerated excitons and unbound e-h plasma by removing the phonon bleaching contributions. This is justified by the facts that the phonon modes in $\Delta\sigma_1(\omega)$ are well-defined local oscillators and only affect the spectral weight near the resonances. The calculated THz response functions (black lines) in Fig. 3a consistently reproduce very well the experimental results, which

are divided into individual components as the intra-excitonic $1s\rightarrow 2p$ (red lines), $1s\rightarrow 3p$ (green lines) and unbound *e-h* carriers (blue lines) . The presence and dynamic evolution of this characteristic THz spectral shape, as shown in Figs. 3b and 3c, provide both qualitative signature and quantitative measure for exciton formation, which have been discussed in Fig. 4 of the main text to be focused next. The distinct spectral shapes of the internal quantum transition of exciton Rydberg states and Drude-Smith carriers allow to faithfully extract their effective transition strength, $A^X_{1s\rightarrow 2p}$ (red dots), $A^X_{1s\rightarrow 3p}$ (green dots) and A^{eh}_{DS} (blue dots) in the sample for the 790 nm excitation at a fixed time delay 60 ps, as shown in Supplementary Figure 5. Here the internal excitonic transitions gradually diminish above the structure phase transition T_S ∼160 K, while the Drude-Smith carriers exhibit an abrupt jump in their transition strength near this temperature. These distinct temperature dependent results fully corroborate our conclusion in the main text that the dielectric screening is significantly enhanced cross the structural phase transition and suppresses the excitonic correlation. Although this has been studied in the near-zero-frequency limit, our results unambiguously establish this missing link in the relevant THz spectral region.

The exciton and free charge carrier densities in our sample are calculated by fitting the integrated spectral weight of extracted THz conductivities and dielectric functions. We understand the accurate estimation of densities in such a highly inhomogeneous medium with randomly dispersed perovskite microcrystals is challenging. In the following we will compare the calculation of carrier densities from 2 different methods, i.e., from (1) THz conductivity and dielectric function and (2) absorbed pump photon density. Theoretically, the two should be very similar. The results show that densities are very similar for 790 nm excitation, and have a little difference for 399 nm excitation, the possible reasons of which will be discussed later. Additionally, we also notice big scattering of visible light from perovskite crystals (Fig. 1e)

in the absorption measurement, as confirmed by another perovskite study[29], which justifies the relatively high pump fluences used against the moderate excited carrier densities calculated.

According to Ref[9], the absorption coefficient of perovskite materials for 790 nm light is $\sim$2,300/cm, corresponding to a penetration depth of 4.35 μm. Our 790 nm excitation fluence 550 $\mu J/cm^2$ (corresponding to $2.19\times10^{15}/cm^2$ photon density) is measured before the low temperature cryostat. After taking into account the 790 nm reflection from cryostat sapphire window (R=14.6%) and PMMA film (R=3.8%), the actual density of photons entering the perovskite/PMMA film is $2.19\times10^{15}/cm^2\times(1\text{-}14.6\%)\times(1\text{-}3.8\%) = 1.8\times10^{15}/cm^2$. Because of the light scattering, a larger portion of pump photons entering the film will be scattered instead of absorbed. We estimate the scattering coefficient by employing the same method as presented by Ref[29], and using the optical density of an individual crystal from this paper as a reference, we get for our sample the perovskite crystal coverage ratio $\approx$1, scattering coefficient $\approx$0.945, and thickness coefficient $\approx$17.35. This implies our sample is almost fully covered with perovskite crystals. Although the filling ratio is only $\sim$1.2%, the perovskite/PMMA film is pretty thick $\sim$550 μm, so it is not surprising that they can fully cover the film where measurement is taken. This also suggests the effective thickness of the perovskite crystals to be 6.6 μm. In addition, scattering coefficient of 0.945 implies 94.5% of the incoming visible light gets scattered and only 5.5% of it is absorbed. This is about twice as large as the scattering coefficient in the thin film case from Ref[29], indicating our dispersed microcrystals is more inhomogeneous than thin film. Because the perovskite penetration depth is smaller than the effective thickness, the former should be used for density calculation. We calculate the absorbed 790 nm pump photon density to be $1.8\times10^{15}/cm^2\times(1\text{-}94.5\%)/4.35\ \mu m = 2.28\times10^{17}/cm^3$. This is similar to the sum of excited exciton and free carrier densities we calculated from THz conductivity and dielectric function, as shown in Figs. 4a and 4c, which is $\sim1.35\times10^{17}/cm^3$.

For 399 nm excitation the penetration depth, according to Ref[9] is $\sim$0.08 μm. The 399 nm fluence of 120 μJ/cm^2 corresponds to 2.4$\times$10^{14}/cm^2 photon density before the cryostat. Similarly, after taking into account the reflection from sapphire window (R=15.3%) and PMMA film (R=4.1%), the actual density of pump photons entering the perovskite/PMMA film is 2.4$\times$10^{14}/cm^2$\times$(1-15.3%)$\times$(1-4.1%)=1.95$\times$10^{14}/cm^2. With penetration depth of 0.08 μm and scattering coefficient of 0.945, we calculate the absorbed 399 nm pump photon density to be 1.34$\times$10^{18}/cm^3. This number represents the upper limit for 399 nm excitation, which is an order of magnitude larger than the total carrier density that we calculated from the THz conductivity and dielectric function ($\sim$1.34$\times$10^{17}/cm^3), as shown in Figs. 4b and 4c. There are two more factors that should be taken into considerations: (1) the scattering coefficient should be wavelength dependent. If a simple Rayleigh scattering of $\sim \lambda^{-4}$ is assumed, the coefficient for 399 nm should be roughly an order of magnitude larger than that for 790 nm, but in our calculation we simply consider it a constant (the same way as the reference paper did). (2) the reported penetration depth of the perovskite materials at 399 nm varies more than an order of magnitude for various literature, which may greatly affect the accuracy of density estimation. For example, a depth of $\sim$30 nm from Ref[8], $\sim$80 nm from Ref[9], and $\sim$400 nm from Ref[38] are reported. Although we do not think the difference is intrinsic of the perovskite materials, we understand there are a lot of fine details, such as measurement technique, scattering, sample phase and preparation processes, etc. which can change the penetration depth measurement from sample to sample.

Given the above 2 reasons, we think the actual absorbed 399 nm photon density should be $\sim$ 1$\times$10^{17}/cm^3, similar to the one we calculated from THz conductivity and dielectric function. Therefore the photoexcited exciton density is below the density where the Auger recombination starts to play a role $\sim$2$\times$10^{18}/cm^3 (Ref[39]).

Another evidence to confirm the validity of our estimation of the carrier densities from THz conductivity and dielectric function is that, as shown in Fig. 4c, the free carrier densities after 790 nm and 399 nm excitations decay in a similar rate, indicating similar excited carrier densities for the 2 pump wavelengths, because as shown in Ref[39], the carrier recombination rate is highly density dependent especially when Auger recombination starts to be effective.

For exciton dynamics, our THz lineshape analysis yields the time evolution of exciton population and carrier density shown in Figs. 4a-4c. In the resonant coherent excitation regime, the intrinsic microscopic pathway for the buildup of exciton population is shown to originate from the dephasing of electronic coherence and scattering with discrete phonons. The polarization-to-population conversion time is shown to be on $\sim$1 ps time scales. In the largely incoherent regime with e-h continuum excitation, cooling of the hot population can be directly accessed based on the fitted, time-evolution of exciton population difference $N_{1s} - N_{2p}$ and $N_{1s} - N_{3p}$ with a given distribution function. The oscillator strength used can be determined theoretically based on the hydrogen model. For $1s \rightarrow \mathrm{n}p$ transition, they can be expressed as

$$f_{1s\rightarrow \mathrm{n}p} = \frac{2^8 \mathrm{n}^5 (\mathrm{n}-1)^{2\mathrm{n}-4}}{3(\mathrm{n}+1)^{2\mathrm{n}+4}}, \tag{5}$$

which gives $f_{1s\rightarrow 2p} = 0.416$ and $f_{1s\rightarrow 3p} = 0.079$. Since excitons are composite bosons, with repulsive interaction to the second order, formed in a fermion many-body systems, one may not simply expect a pure Bose-Einstein or rigorous Fermi-Dirac distribution functions of hot excitons. Assuming that quasi-equilibrium has been reached at early stage and the Fermi-Dirac distribution, we further extract the effective temperature T^* evolution of the excitons using the relation

$$\frac{N_{1s} - N_{2p}}{N_{1s} - N_{3p}} = \frac{\int D_{1s}(\omega) f(\omega) d\omega - \int D_{2p}(\omega) f(\omega) d\omega}{\int D_{1s}(\omega) f(\omega) d\omega - \int D_{3p}(\omega) f(\omega) d\omega} \tag{6}$$

where the density of states of the ns/p excitons in the three-dimensional space is given by

$$D_{\mathrm{n}} \propto \sqrt{\omega - \omega_{\mathrm{n}}}. \tag{7}$$

Given the experimentally-extracted ratio $\Delta N_{1s,2p}/\Delta N_{1s,3p}$ (inset, Fig. 4d), we calculate the effective temperature T^* using the above relations. Fig. 4d in the main text shows that excitons stimulated by the 399 nm pulses are initially at quite high temperature, and gradually cool down on 10s of ps time scale. Meanwhile, the excitons generated by the 790 nm pulses are already very cool, close to the lattice temperature at the early stage. Finally, Fig. 4c shows that the photoexcitation quasi-instantaneously convert into mobile carriers, despite the absence of longer range transport hindered by disorder and/or crystal boundary as seen in the suppressed $\Delta\sigma_1(\omega)$ as $\omega \to 0$.

5 Supplementary Note 5: Further discussions for delayed exciton buildup with below-resonance pumping

The complete characterization of full response functions of both excitons and charge carriers has not been possible in prior measurements in this system. The response functions observed here directly demonstrate, without relying on assumptions and models, that there exists a slow buildup of the internal quantum transitions under below resonance pumping. There are two main processes that contribute to delayed exciton buildup when the pump excites below the exciton peak seen in linear absorption, as seen in Fig. 4a and 4e. First, the laser field couples to coherent e–h excitation amplitudes with zero center-of-mass (CM) momentum when momentum is a good quantum number. In the case of strong disorder or phonon shakeup processes, a distribution of finite CM momentum excitons is excited. As a second step, scattering processes, mainly with phonons, transform such coherent e–h pair excitations into incoherent electron and hole populations with different momenta or redistribute the photoexcited excitons

among different CM states along the $1s$ parabola. Such populations resulting from exciton-phonon scattering grow as the e–h polarization dephases following laser excitation. Therefore, initially the build-up of low-energy $1s$ exciton populations occurs on a timescale determined by the optical polarization dephasing time, which arises from multiple scattering contributions and disorder effects, while later it is dominated by scattering with discrete or continuum phonons. The linewidths of the observed peaks in both absorption and THz measurements indicate polarization dephasing times of the order of 1 ps, which explains the initial rise-time shown in Fig. 4e. The nearly ps exciton formation time, determined by the conversion of electronic coherence, are further supported by a recent four-mixing experiment showing dephasing time times of ∼600 fs at 10K, which is largely consistent with our results and interpretation [?]. Following dephasing of the laser-driven exciton polarization, higher CM momentum states in the $1s$ exciton parabola become populated. Subsequently, these exciton populations relax towards the lowest energy states with small momenta by scattering with phonons. Unlike in GaAs and other inorganic semiconductors, here we observe discrete phonon states with energies on the order of 2-8 meV, i.e. in the few THz range, as shown in Figs. 2b and 2d. Such phonon energies are comparable to the exciton CM kinetic energies. Similar to optical phonons in GaAs, yet at much higher energy ∼36 meV, here the presence of discrete THz phonon states in the perovskite materials can lead to carrier and exciton relaxation on a timescale of few ps, much faster than scattering with acoustic phonons for near–resonance pumping [2]. For example, excitons with large CM momentum can lose finite kinetic energy by a single scattering event with a discrete phonon, which absorbs the CM momentum. We thus attribute the observed rise time in Figs. 4a and 4e to the loss of exciton coherence, τ^{fast} ∼1.0±0.03 ps, and the phonon-assisted processes that involve the low energy discrete phonon states in our system, τ^{slow} ∼11.2±1.06 ps at 8 K, which lead to a redistribution of exciton states with different CM momenta along the $1s$ parabola.

Supplementary References

1. Kira, M. & Koch, S. W. Many-body correlations and excitonic effects in semiconductor spectroscopy. *Prog. Quan. Elec.* **30**, 155-296 (2006).

2. Siantidis, K., Axt, V. M. & Kuhn T. Dynamics of exciton formation for near band-gap excitations. *Phys. Rev. B* **65**, 035303 (2001).

3. Kaake, L. G., Moses, D. & Heeger, A. J. Coherence and Uncertainty in Nanostructured Organic Photovoltaics. *J. Phys. Chem. Lett.* **4**, 2264-2268 (2013).

4. Falke, S. M. *et al.* Coherent ultrafast charge transfer in an organic photovoltaic blend. *Science* **344**, 1001-1005 (2014).

5. Bakulin, A. A. *et al.* The Role of Driving Energy and Delocalized States for Charge Separation in Organic Semiconductors. *Science* **335**, 1340-1344 (2012).

6. Saidaminov, M. I. *et al.* High-quality bulk hybrid perovskite single crystals within minutes by inverse temperature crystallization. *Nat. Commun.* **6**, 7586 (2015).

7. Zhu, F. *et al.* Shape Evolution and Single Particle Luminescence of Organometal Halide Perovskite Nanocrystals. *ACS Nano* **9**, 2948-2959 (2015).

8. Green, M. A., Ho-Baillie, A. & Snaith, H. J. The emergence of perovskite solar cells. *Nat. Photon.* **8**, 506-514 (2014).

9. Xing, G. *et al.* Long-Range Balanced Electron and Hole-Transport Lengths in Organic-Inorganic $CH_3NH_3PbI_3$. *Science* **342**, 344-347 (2013).

10. Yang, W. S. *et al.* High-performance photovoltaic perovskite layers fabricated through intramolecular exchange. *Science* **348**, 1234-1237 (2015).

11. Stranks, S. D. *et al.* Electron-Hole Diffusion Lengths Exceeding 1 Micrometer in an Organometal Trihalide Perovskite Absorber. *Science* **342**, 341-344 (2013).

12. D′Innocenzo, V. *et al.* Excitons versus free charges in organo-lead tri-halide perovskites. *Nat. Commun.* **5**, 3586 (2014).

13. Herz, L. M. Charge-Carrier Dynamics in Organic-Inorganic Metal Halide Perovskites. *Annu. Rev. Phys. Chem.* **67**, 65-89 (2016) and references therein.

14. Valverde-Chavez, D. A. *et al.*, Intrinsic femtosecond charge generation dynamics in single crystal $CH_3NH_3PbI_3$. *Energy Environ. Sci.*, **8**, 3700-3707 (2015).

15. Miyata, A. *et al.* Direct measurement of the exciton binding energy and effective masses for charge carriers in organicinorganic tri-halide perovskites. *Nat. Phys.* **11**, 582-587 (2015).

16. Savenije, T. J. *et al.* Thermally Activated Exciton Dissociation and Recombination Control the Carrier Dynamics in Organometal Halide Perovskite. *J. Phys. Chem. Lett.* **5**, 2189-2194 (2014).

17. Tilchin, J. *et al.* Hydrogen-like Wannier-Mott Excitons in Single Crystal of Methylammonium Lead Bromide Perovskite. *ACS Nano* **10**, 6363-6371 (2016).

18. Kong, W. *et al.* Characterization of an abnormal photoluminescence behavior upon crystal-phase transition of perovskite $CH_3NH_3PbI_3$. *Phys. Chem. Chem. Phys.* **17**, 16405-16411 (2015).

19. Even, J., Pedesseau, L. & Katan, C. Analysis of Multivalley and Multibandgap Absorption and Enhancement of Free Carriers Related to Exciton Screening in Hybrid Perovskites. *J. Phys. Chem. C* **118**, 11566-11572 (2014).

20. Lin, Q., Armin, A., Nagiri, R. C. R., Burn, P. L. & Meredith, P. Electro-optics of perovskite solar cells. *Nat. Photon.* **9**, 106-112 (2015).

21. Kaindl, R. A., Carnahan, M. A., Hägele, D., Lövenich, R. & Chemla, D. S. Ultrafast terahertz probes of transient conducting and insulating phases in an electronhole gas. *Nature* **423**, 734-738 (2003).

22. Luo, L. *et al.* Broadband terahertz generation from metamaterials. *Nat. Commun.* **5**, 3055 (2014).

23. Chatzakis, I. *et al.* Reversible modulation and ultrafast dynamics of terahertz resonances in strongly photoexcited metamaterials. *Phys. Rev. B* **86**, 125110 (2012).

24. La-o-vorakiat, C. *et al.* Elucidating the role of disorder and free-carrier recombination kinetics in $CH_3NH_3PbI_3$ perovskite films. *Nat. Commun.* **6**, 7903 (2015).

25. La-o-vorakiat, C. *et al.* Phonon Mode Transformation Across the Orthohombic-Tetragonal Phase Transition in a Lead Iodide Perovskite $CH_3NH_3PbI_3$: A Terahertz Time-Domain Spectroscopy Approach. *J. Phys. Chem. Lett.* **7**, 1-6 (2016).

26. Karakus, M. *et al.* Phonon-Electron Scattering Limits Free Charge Mobility in Methylammonium Lead Iodide Perovskites. *J. Phys. Chem. Lett.* **6**, 4991-4996 (2015).

27. Milot, R. L., Eperon, G. E., Snaith, H. J., Johnston, M. B. & Herz, L. M. Temperature-Dependent Charge-Carrier Dynamics in $CH_3NH_3PbI_3$ Perovskite Thin Films. *Adv. Funct. Mater.* **25**, 6218-6227 (2015).

28. Onoda-Yamamuro, N., Matsuo, T. & Suga, H. Dielectric study of $CH_3NH_3PbX_3$ (X=Cl, Br, I). *J. Phys. Chem. Solids.* **53**, 935-939 (1992).

29. Tian, Y. & Scheblykin, I. G. Artifacts in Absorption Measurements of Organometal Halide Perovskite Materials: What Are the Real Spectra? *J. Phys. Chem. Lett.* **6**, 3466-3470 (2015).

30. Wang, J., Khodaparast, G.A., Kono, J., Oiwa, & A., Munekata, H., Ultrafast optical and magneto-optical studies of III-V ferromagnetic semiconductors. *J. Mod. Opt.* **51**, 2771-2780 (2009).

31. Luo, L., Chatzakis, I., Patz, A. & Wang, J. Ultrafast Terahertz Probes of Interacting Dark Excitons in Chirality-Specific Semiconducting Single-Walled Carbon Nanotubes. *Phys. Rev. Lett.* **114**, 107402 (2015).

32. Richter, C. & Schmuttenmaer, C. A. Exciton-like trap states limit electron mobility in TiO_2 nanotubes. *Nat. Nanotech.* **5**, 769-772 (2010).

33. Smith, N. V. Classical generalization of the Drude formula for the optical conductivity. *Phys. Rev. B* **64**, 155106 (2001).

34. Yang, Y. *et al.* Observation of a hot-phonon bottleneck in lead-iodide perovskites. *Nat. Photon.* **10**, 53-59 (2016).

35. March, S. A., Clegg, C., Riley, D. B., Webber, D., Hill, I. G., Hall, K. C., Simultaneous observation of free and defect-bound excitons in $CH_3NH_3PbI_3$ using four-wave mixing spectroscopy. Preprint at http://arxiv.org/abs/1608.02019 (2016).

36. Umari, P., Mosconi, E. & De Angelis, F. Relativistic GW calculations on $CH_3NH_3PbI_3$ and $CH_3NH_3SnI_3$ Perovskites for Solar Cell Applications *Sci. Rep.* **4**, 4467 (2014).

37. Quintavalle, D. *et al.* Structure and properties of the stable two-dimensional conducting polymer Mg_5C_{60}. *Phys. Rev. B* **77**, 155431 (2008).

38. Park, N. Perovskite solar cells: an emerging photovoltaic technology. *Mater. Today* **18**, 65-72 (2015).

39. Yang, Y. *et al.* Comparison of Recombination Dynamics in $CH_3NH_3PbBr_3$ and $CH_3NH_3PbI_3$ Perovskite Films: Influence of Exciton Binding Energy. *J. Phys. Chem. Lett.* **6**, 4688-4692 (2015).
